# Proposal of New Key Step in Lateral Approach Thyroidectomy in Light of Comparison of Surgical Outcomes of Medial versus Lateral Approach Thyroidectomy: A Randomised Controlled Study

**DOI:** 10.1155/2021/8546860

**Published:** 2021-03-04

**Authors:** Jyotirmoy Phookan, Shilpi Gupta, Nabajyoti Saikia, Debajit Sarma, Mohan Kumar Mili, Mridusmita Gohain, Joydeep Dey

**Affiliations:** ^1^Department of Otorhinolaryngology, Assam Medical College, Dibrugarh, Assam, India; ^2^Department of Otorhinolaryngology, Gauhati Medical College and Hospital, Gauhati, Assam, India; ^3^Department of Otorhinolaryngology, Diphu Medical College and Hospital, Diphu, Assam, India

## Abstract

**Objective:**

Thyroid surgery has travelled a long path, from a surgery which once was considered deemed to fail and even led to death, to the current time when newer techniques are being tested to make the incision smaller and smaller. The aim of our study is to give a precise detailed stepwise description of medial and lateral surgical approach with the anatomical basis and to compare their feasibility and safety.

**Methods:**

104 cases presenting with thyroid swelling in the Department of Otorhinolaryngology, Assam Medical College Dibrugarh from January 1, 2019, to December 31, 2019, were selected and randomisation was done based on day of admission from OPD. Basic demographic data, preoperative diagnosis, operative time, blood loss, need for transection of strap muscles, and complications were recorded.

**Result:**

The distribution of thyroid cases according to age ranged from 17 to 81 years. The prevalence of thyroid disorders was the highest (37.5%) in the age group of 31–40 yrs. Of a total of 104 thyroid cases, 49 were colloid goitre, 24 were multinodular goitre, 9 were follicular neoplasm category 4, 4 were papillary thyroid carcinoma, 2 were follicular neoplasm category 3, and 3 were follicular neoplasm category 5.

**Conclusion:**

Out of the two approaches, lateral approach thyroidectomy showed better results with fewer complications. A single structure (superior belly of omohyoid) can be used as a guide to preserving all vital structures related to the thyroid gland.

## 1. Introduction

The ambitions of a thyroid surgeon have evolved along the timeline. In 1866 Samuel Gross wrote: “*Can the thyroid in the state of enlargement be removed? Emphatically, experience answers no. Should the surgeon be so foolhardy to undertake it....every stroke of the knife will be followed by a torrent of blood and lucky it would be for him if his victim lived long enough for him to finish his horrid butchery. No honest and sensible surgeon would ever engage in it.*”

Thyroid surgery has travelled a long path since then, from a surgery that was once doomed to fail and even led to the death of the patient, to the present time when newer techniques are being tested to make the incision smaller and smaller. In this study, we have compared medial and lateral approach thyroidectomy and identified vital structures with the help of superior belly of omohyoid like superior laryngeal nerve, middle thyroid vein, recurrent laryngeal nerves, parathyroid glands with least complication, and least long-term morbidity.

The conventional thyroidectomy with the midline approach (MA) is started with midline splitting of strap muscles and retracting them laterally. The three vital elements of thyroid anatomy, namely, the vessels, the recurrent laryngeal nerves (RLN), and the parathyroid glands, are identified. The vessels are ligated while the RLN and parathyroid lands are preserved followed by the mobilisation of the thyroid.

In the lateral approach (LA) thyroidectomy, we propose a triangle bounded by the superior belly of omohyoid laterally, the strap muscles in the midline and thyroid lobe forming the base. Through this triangle, we can manage the superior thyroid vessels, superior parathyroids, the middle thyroid vein, and recurrent laryngeal nerve and easily start the lateral neck dissection (if needed) without splitting any extra fascia, therefore facilitating easy mobilisation and delivery of thyroid swelling without division or excessive retraction of strap muscles.

In this study, we aimed at precisely describing the surgical technique of both approaches with their anatomical basis and presenting the preliminary results to evaluate feasibility and safety in light of operative time, blood loss, need for transection of strap muscles, and complications associated with vital structures involved in thyroidectomy.

## 2. Materials and Methods

**Figure 1:**
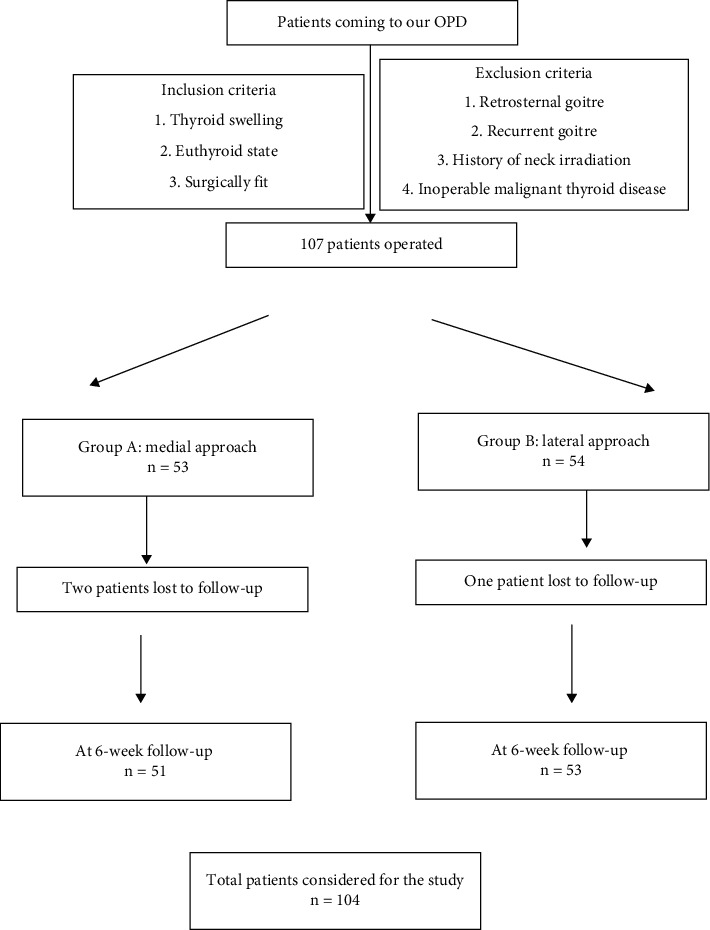
The study design of this randomised controlled trial.

The study was conducted from the 1^st^ January 2019 to 31^st^ December 2019 in the Department of Otorhinolaryngology in a tertiary care center of Assam, India. A total of 104 patients were included, operated, and followed up (Figure 1). The patients were selected based on our inclusion criteria, that is, all the patients coming to our OPD with thyroid swelling in an euthyroid state, surgically fit patients. Our exclusion criteria were retrosternal goitre, recurrent goitre, history of neck irradiation, and inoperable malignant thyroid disease (TNM staging).

A total of 51 patients underwent MA and 53 LA thyroidectomy based on randomisation according to the day of admission from our out-patient department. Cases admitted on Monday, Wednesday, and Friday were put in group A, that is, the MA group, while patients admitted on Tuesday, Thursday, and Saturday were put in group B, that is, the LA group. Preoperative checkup included proper history, clinical examination, thyroid profile, ultrasound, FNAC, CT neck and thorax (if needed), video direct laryngoscopy to check vocal cord movement, and preoperative serum calcium measurement. Preoperative anaesthesia clearance was taken after ruling out any comorbid condition.

The statistical analysis of data was performed using the computer program, Statistical Package for Social Sciences (SPSS for Windows, version 20.0. Chicago, SPSS Inc.) and Microsoft Excel 2010. Results on continuous measurements are presented as mean ± standard deviation and are compared using Students' *t*-test. Discrete data are expressed as number (%) and are analysed using Chi square test and Fischer's exact test (where the cell counts were <5 or 0). Pearson's correlation coefficient (*r*) was used to measure the associations among continuous variables. For all analyses, the statistical significance was fixed at a 5% level (*p* value < 0.05).

Before commencing the study, necessary permission and approval from the ethics committee were obtained from the Institutional Ethics Committee (Human), Assam Medical College and Hospital (636). Informed written consent was obtained from all the patients involved in the study according to the protocol approved by the Ethics Committee and after explaining the procedure to them in their own understandable language. All the operations were done by the same surgical team.

Basic demographic data, preoperative diagnosis, type of surgery done, operative time, blood loss, complications, the need to divide strap muscles, identification of parathyroid glands, recurrent laryngeal nerves and superior laryngeal nerve, and postoperative complications were recorded.

Under general endotracheal anaesthesia, the patients were kept in a supine position with gentle neck extension and arms stuck to the patient's both sides with perfect alignment of the head and body for suitable placement of the incision and adequate anatomical exposure. In both techniques, the same 4–6 cm skin crease neck incision was made 2 cm above the suprasternal notch. Superior and inferior subplatysmal flaps were raised. The anterior branch of the jugular vein was preserved in all cases.

**Figure 2:**
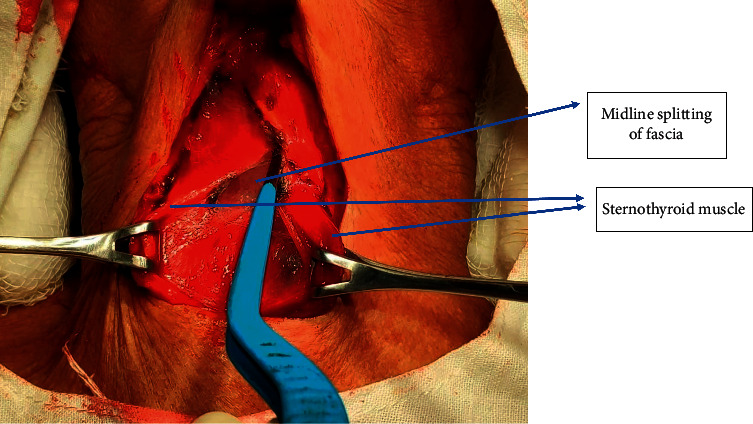
Midline separation of fascia in the medial approach.

**Figure 3:**
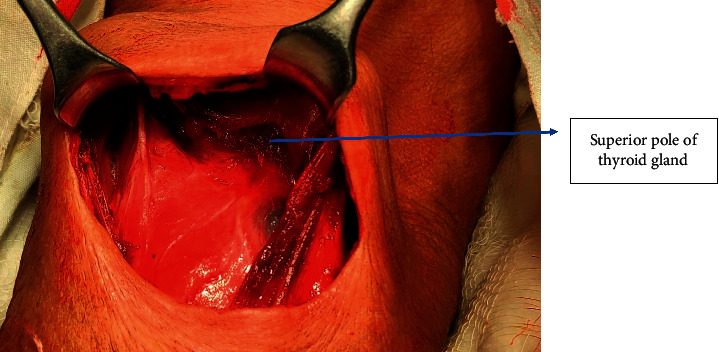
Strap muscles retracted laterally in the medial approach.

In MA, midline separation of fascia was done followed by retraction of strap muscles laterally as shown in Figures 2 and 3. For exposure of bigger thyroid swelling, the strap muscles were sacrificed. The superior pole of the thyroid gland was identified by retracting the thyroid gland downwards. Vessels of the superior pedicle and middle thyroid vein were individually ligated. Then, an external branch of the superior laryngeal nerve (SLN) and RLN (identified at the cricothyroid junction) was preserved. Superior and inferior parathyroid glands were identified and preserved. The inferior thyroid vein was ligated and cut. The thyroid isthmus was separated from Berry's Ligament and underlying tracheal rings.

In LA, instead of midline separation and retraction of strap muscles, the anterior border of Sternocleidomastoid (SCM) was identified and mobilized laterally. The superior belly of the omohyoid is traced up to its insertion in the hyoid bone and retracted cranially. The lateral edge of strap muscles was identified and retracted medially to expose the underlying goitre.

**Figure 4:**
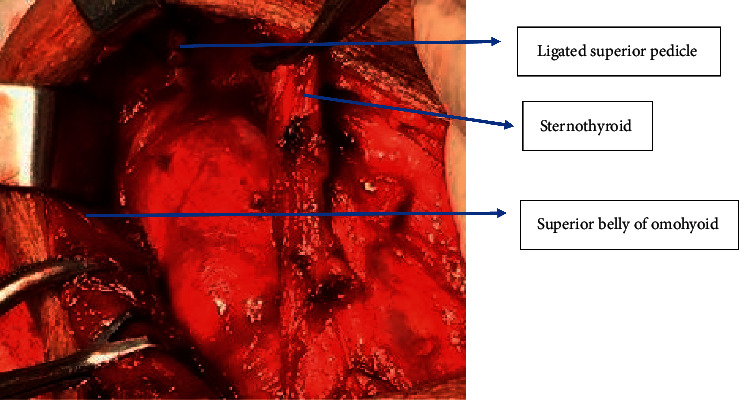
Exposure of superior pedicle of right thyroid lobe via the lateral approach.

**Figure 5:**
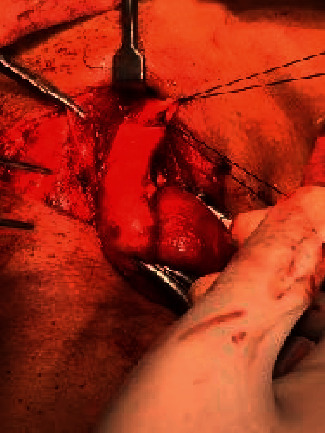
Ligation of superior pedicle via the lateral approach.

**Figure 6:**
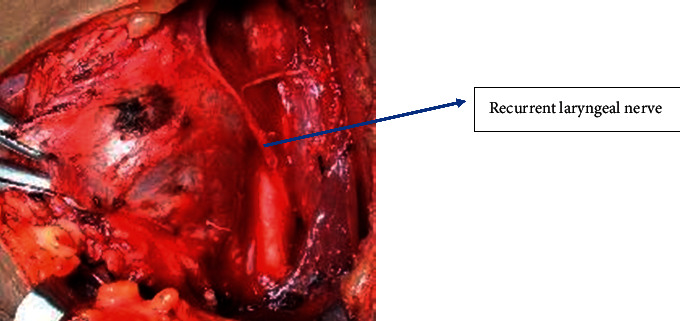
Identification of recurrent laryngeal nerve.

**Figure 7:**
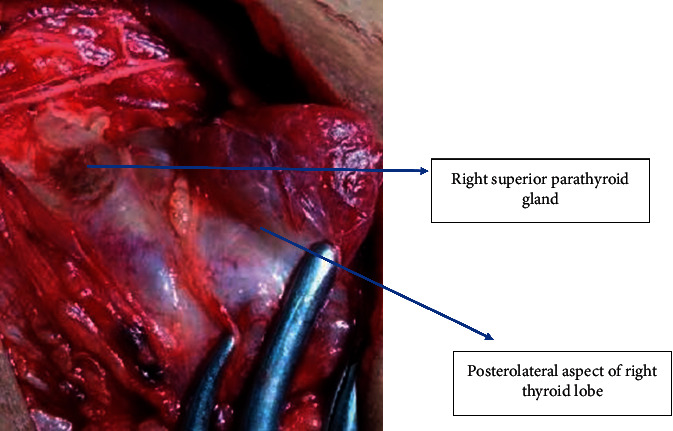
Identification of superior parathyroid gland.

Next, the thyroid was retracted downwards and the superior pole of the thyroid was identified in the triangle as shown in Figure 4. The superior vascular pedicle could be easily identified by this technique allowing individual ligation of vessels as shown in Figure 5 and preservation of external laryngeal nerve. Once the middle thyroid vein was identified (if present) mostly at the junction of the superior belly of omohyoid and internal jugular vein and ligated, the rest of the thyroid lobe can be easily dissected and retracted medially for easier identification of recurrent laryngeal nerve at the point of entry at the cricothyroid junction or in Simon's triangle as shown in Figure 6. The superior parathyroid gland is identified in the lateral triangle itself in the posterolateral aspect of the thyroid lobe as shown in Figure 7 and the inferior parathyroid is found in close proximity to inferior thyroid vessels. The inferior thyroid veins can be easily located travelling transversely over the common carotid artery and are ligated. The thyroid lobe is freed from its attachment to the ligament of Berry and underlying trachea rings.

**Figure 8:**
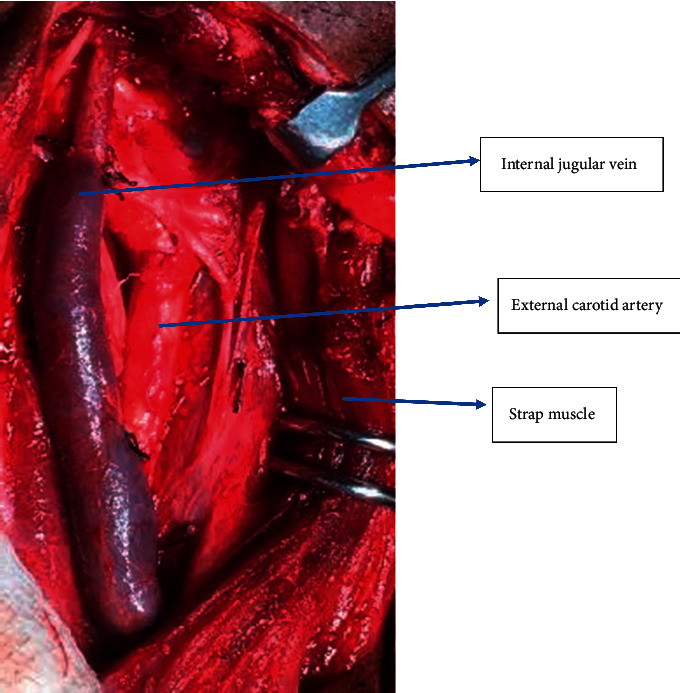
Lateral neck dissection (all lymph nodes cleared).

**Figure 9:**
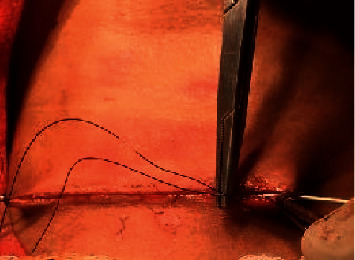
Subcuticle closure.

In both techniques, for hemithyroidectomy, the isthmus is then transected to complete the procedure. For patients undergoing total thyroidectomy, a similar dissection of the opposite lobe is performed. Once both the thyroid lobes were fully dissected and freed from the overlying strap muscles, the smaller lobe could be easily pushed beneath strap muscles to the opposite side. With LA, lateral neck dissection can be carried out without any extra incision in the deep fascia as shown in Figure 8. A warm normal saline wash is given and hemostasis is achieved by ligation and cauterization. At the end of the surgery, the midline strap muscles are approximated and deep fascia is sutured. A drain is given in all the cases with MA and is omitted in cases operated with LA. The wound is closed in layers and subcuticular skin sutures are given as shown in Figure 9. All total thyroidectomy specimens were removed intact in both the group and were measured and sent for histopathological examination.

Intraoperative blood loss was calculated by the total of drain collection and number of gauzes used (1 gauze = 7 ml blood).

Postoperative voice changes, swallowing difficulty, postoperative stridor or respiratory distress, tetany (both by trousseau sign and serum calcium level measured after 24 hrs), hematoma, or serosa formation were checked clinically.

Patients were discharged on the 3^rd^ postoperative day with or without thyroxine (depending upon the procedure). Follow-up was done after 1, 2, and 6 weeks. Healing of scar mark and TSH level and other comorbidities was noted.

## 3. Results

The current study is a randomised controlled trial carried out for a duration of 1 year from January 1, 2019, to December 31, 2019, involving 104 patients, out of which 91 were females and 13 males with a sex ratio (female: male) of 7 : 1.

The distribution of thyroid cases according to age ranged from 17 to 81 years. Of total thyroid cases, 7 patients were between 11 and 20 years, 20 patients were 21 and 30 years, 39 patients were 31 and 40 years, 25 patients were 41 and 50 years, 8 patients were 51 and 60 years, 2 patients were 61 and 70 years, and 3 patients were above 70 yrs. The prevalence of thyroid disorders was the highest (37.5%) in the age group of 31–40 yrs. The distribution of thyroid disorders is summarised in Table 1. The mean ages of patients in the medial approach group and lateral approach group are 39.64 ± 12.30 and 38.26 ± 13.29, respectively.

Out of total 104 thyroid cases, 49 were colloid goitre, 24 were multinodular goitre, 9 were follicular neoplasm cat 4, 4 were papillary thyroid carcinoma, 2 were follicular neoplasm cat 3, 3 were follicular neoplasm category 5, 6 were follicular neoplasm category 2, 2 were medullary thyroid cancer, 2 were undifferentiated cancer, 2 were hurtle cell neoplasm, and 1 was granulomatous thyroiditis. A total of 91 patients were euthyroid; 13 were controlled toxic goitre.

Out of a total of 104 cases, 51 patients underwent MA and 53 LA thyroidectomy based on randomisation according to the day of admission from OPD (Table 2).

Table 3 shows the procedure performed in 104 cases. In 69 cases, hemithyroidectomy was done; in 23 cases, total thyroidectomy alone was done; 10 cases had total thyroidectomy with neck dissection; 1 case had isthmectomy; and another 1 had completion thyroidectomy.

Parathyroid glands and recurrent laryngeal nerve were identified and preserved in all patients of LA. The external branch of the superior laryngeal nerve was identified and preserved in all cases with LA. With MA, in 2 cases of parathyroid glands, 5 cases of RLN and 5 cases of the external branch of SLN were not identified. For LA, the mean incision length (cm) was 4.46 ± 0.498, the amount of intraoperative blood loss (ml) was 88.30 ± 22.12, and the mean operative time (min) was 93.20 ± 21.39. For MA, the mean incision length (cm) was 4.47 ± 0.48, the amount of intraoperative blood loss (ml) was 89.50 ± 23.36, and the mean operative time (min) was 96.76 ± 22.06. The mean hospital stay for LA and MA approaches is 3.86 ± 1.22 and 4.28 ± 1.37. We did not encounter any intraoperative or postoperative mortality. The difference in incision length, operating time, blood loss, and hospital stay between the two procedures was statistically insignificant (*p* value>0.05). However, there were fewer complications in the LA group as compared to the MA group.  Table 4 shows both intraoperative and postoperative complications associated with both approaches.

## 4. Discussion

“The extirpation of the thyroid gland for goitre typifies perhaps better than any other operation the supreme triumph of the surgeon's art.” William Halsted (1852–1922).

Thyroid pathology has been known since ancient times, and surgical management of thyroid diseases, however, evolved slowly throughout the ages. Currently, open thyroidectomies are typically performed through a 4–6 cm transverse incision made in the anterior lower neck. Towards the end of the twentieth century, new techniques for thyroidectomy were developed to include both minimally invasive and extracervical remote-access surgery. Minimally Invasive Thyroidectomy (MIT) or Minimally Invasive Procedure (MIP) should properly be defined as operations through a short and discrete incision that permits direct access to the thyroid or parathyroid gland, resulting in a focused dissection [1]. The aim of our study is to give a precise detailed stepwise description of the medial and lateral surgical approach and its anatomical basis and to compare their feasibility and safety.

In our study, there was no significant difference in patient's demographics and type of thyroid surgery in each group. The distribution of thyroid cases according to age ranged from 17 to 81 years, which is depicted in Table 1. The prevalence of thyroid disorders was the highest (37.5%) in the age group of 31–40 yrs.

Tomimori et al. in their study showed different pathological reporting in thyroid disease with combined ultrasonographic and cytological studies concluding that using an index derived from combined ultrasonographic and cytologic studies will result in a better patient selection for surgery [2]. In our study, the distribution of thyroid disorder according to their diagnosis based on USG findings and FNAC findings is summarised in Table 1. Of a total of 104 thyroid cases, benign lesions were 89.4% and 10.6% of cases were either suspicious nodule or confirmed malignancy. The most common diagnosis was colloid goitre accounting for 47.1% euthyroid; 13 were controlled toxic goitre.

In a previous study (by author on 30 cases), we described a single compartment surgery that is the vascular cases and the least common diagnosis was thyroid malignancy accounting for 7.6% of cases. 91 patients were compartment and defined a new landmark triangle for the external branch of SLN and superior thyroid pedicle in between the omohyoid, sternothyroid muscle, and upper pole of the thyroid as the lower border, that is, the triangle of sternothyroid omohyoid (of JP) [3]. In this study, we elaborated our step in LA by including 104 cases and compared it with MA.

In LA, by using our proposed triangle, one can easily locate the superior pedicle with the help of the superior belly of the omohyoid. This triangle also harbours external laryngeal nerve which can be easily preserved; the middle thyroid vein can also be identified (if present) mostly at the junction of the superior belly of omohyoid and internal jugular vein and ligated. By retracting the thyroid lobe, medial identification of RLN can be done at the point of entry at the cricothyroid junction in this triangle itself. Superior parathyroid glands in the posterolateral aspect of the thyroid lobe can also be located in this triangle. In cases of thyroid malignancy, lateral compartment neck dissection of lymph nodes can also be carried out with this approach without cutting any extra fascia.

Palazzo et al. in their study of endoscopic thyroidectomy via the LA concluded that their mean operating time was 99 min (range, 64–150 min) [4]. In our study with LA, the mean operative time was 93.20 ± 21.39 minutes. The mean amount of intraoperative blood loss was about 88.30 ± 22.12. Although no drain was given in cases with LA, none of the cases developed a postoperative hematoma and seroma suggesting that this approach helps in meticulous dissection and easy achievement of hemostasis.

LA thyroidectomy succeeded in all study cases with no conversion to strap muscle cutting or sacrificing of the superior belly of omohyoid. Wagner and Seiler in their study showed that a 1.1% risk of RLN palsy is associated with total thyroidectomy [5]. In our study, in LA, voice change was seen in 1 case which improved after steroid administration (0.9% risk). One case developed postoperative stridor due to long-standing goitre with tracheal indentation. Twenty cases developed hypothyroidism after total thyroidectomy.

In MA, midline separation and retraction of strap muscles were done. In some cases, strap muscles were cut for proper exposure of the thyroid gland. Reeve et al. in their study described various complications associated with thyroidectomy and concluded that failure to observe cardinal surgical principles may result in legal difficulties, which can be avoided [6]. In our study, out of 51 MA thyroidectomy patients, 3 cases needed conversion to strap muscle cutting. Voice change was seen in 8 cases which improved after steroid administration. One case developed postoperative stridor. Three cases developed tetany after total thyroidectomy who were administered with calcium supplements. Three cases developed postoperative seroma which improved after giving 7 days of i/v antibiotics. In spite of giving drain in all the cases of MA, 3 cases developed postoperative seroma which can be accounted due to the midline reapproximation of the strap muscles and the lack of space between the SCM and lateral edge of the strap muscles may allow a life-threatening postoperative hematoma or serum beneath the strap muscles while, in LA, no incidence of seroma or hematoma was noted due to a potential gap laterally. One case developed postoperative difficulty in swallowing which improved after 3 months of follow-up. Fourteen cases developed hypothyroidism after total thyroidectomy.

Zambudio et al. in their study showed that, out of 62 patients (21%), corresponding to 29 hypoparathyroidisms, 26 had recurrent laryngeal nerve injuries, 4 lesions of the superior laryngeal nerve, and 3 cervical hematomas [7]. In our study, Table 4 shows the distribution of complications associated with two approaches: in LA minimal, complications were seen, while, in MA, 2.8% of patients needed strap muscle sacrifice, parathyroid injury, and seroma formation. 7.6% of patients showed temporary voice change which improved in all cases after steroid administration. 0.9% of cases showed postoperative swallowing difficulty which improved after 7 days.

For MA, the mean incision length (cm) was 4.47 ± 0.48, the amount of intraoperative blood loss (ml) was 89.50 ± 23.36, and the mean operative time (min) was 96.76 ± 22.06. The mean hospital stay for the LA and MA approaches is 3.86 ± 1.22 and 4.28 ± 1.37. None of the approaches encountered intraoperative or postoperative mortality. The strap muscles play an important role in voice pitch control and swallowing function and hence unnecessary midline division and suturing back of these muscles as done in traditional MA can be avoided using LA.

Hong et al. confirmed the role of strap muscles in phonation using in vivo canine laryngeal model. They proved that handling of strap muscles and reconstitution or even excessive retraction may affect voice quality and swallowing function after thyroidectomy [8].

The excessive retraction of strap muscles laterally in MA, in order to perform dissection posterolaterally along the thyroid lobe, could also be the possible reason for increased postoperative morbidity. Kim et al. compared voice between strap muscle retraction and cutting technique in thyroidectomy and came to the same conclusion [9].

In our study, we got similar results as, in the LA approach, there was a reduced need for strap muscles retraction or transection was found; hence, lower postoperative voice quality loss was seen.

Mohamed et al. in their study summarised that a number of surgeons today have adapted new surgical techniques for thyroid surgery. Even though conventional thyroidectomy remains in the gold standard for thyroidectomy with low morbidity and excellent outcomes, minimally invasive and remote-access techniques have been used in an attempt to avoid visible neck scars without compromising patient's safety and the effectiveness of the procedure [10]. In our study, after follow-up on the 6^th^ week, both approaches showed excellent results in terms of cosmesis of the surgical scar.

Our study has a few limitations like a small sample size and the follow-up was done by the same surgical team and not by any neutral observer. Long-term follow-up could not be done due to challenges during the COVID-19 pandemic. In consensus with our results, a larger study can be performed to prove the advantages of LA over MA.

## 5. Conclusion

In comparison to the medial approach, the lateral approach of thyroidectomy when used by following the superior belly of the omohyoid is a quicker and more precise way of removing thyroid swelling with the least associated complications. It ensures easy identification of structures like superior thyroid pedicle, external branch of superior laryngeal nerve, recurrent laryngeal nerve, middle thyroid vein, and superior parathyroid and also helps in lateral neck dissection without any extra incision. We recommend more widespread use of the lateral approach by considering the identification of the superior belly of the omohyoid as a key step.

## Figures and Tables

**Table 1:** Agewise distribution of preoperative diagnosis.

Preop diagnosis	11–20 yrs	21–30 yrs	31–40 yrs	41–50 yrs	51–60 yrs	61–70 yrs	71–80 yrs	81–90 yrs	Total cases
Colloid goitre	2	9 + 1 (male)	21	12	1 + 1 (male)	—	—	1	49
Multinodular goitre	3	4 + 1 (male)	8	6	1 + 1 (male)	—	—	—	24
Follicular neoplasm cat. 4	1	2	—	2 males	1 male	1 male	2 males	—	9
Papillary carcinoma thyroid	—	3	1 male	—	—	—	—	—	4
Follicular neoplasm cat. 5	—	—	1	1	—	1 male	—	—	3
Follicular neoplasm cat. 3	1	—	2	—	—	—	—	—	2
Follicular neoplasm cat. 2	—	—	4	1	1	—	—	—	6
Medullary ca. thyroid	—	—	—	1	1	—	—	—	2
Undifferentiated ca.	—	—	—	1	1	—	—	—	2
Hurthle cell neoplasm cat. 3	—	—	2	—	—	—	—	—	2
Granulomatous thyroiditis	—	—	—	1	—	—	—	—	1

**Table 2:** The approach used in thyroidectomy.

Pre-op diagnosis	Medial approach	Lateral approach
Colloid goitre	27	22
Multinodular goitre	13	11
Follicular neoplasm cat. 4	2	7
Papillary carcinoma thyroid	—	4
Follicular neoplasm cat. 5	1	2
Follicular neoplasm cat. 3	1	1
Follicular neoplasm cat. 2	3	3
Medullary ca thyroid	1	1
Undifferentiated ca.	1	1
Hurthle cell neoplasm cat. 3	1	1
Granulomatous thyroiditis	1	—

**Table 3:** Preoperative diagnosiswise procedure performed.

Preop diagnostics	Hemithyroidectomy	Total thyroidectomy	Total thyroidectomy with central neck dissection	Total thyroidectomy with central neck and lateral neck dissection	Isthmusectomy	Completion thyroidectomy with central neck dissection and lateral neck dissection
Colloid goitre	42	6 (b/l lobe involved)	—	—	1	—
Multinodular goitre	7	17	—	—	—	—
Follicular neoplasm cat. 4	9	—	—	—	—	—
Papillary carcinoma thyroid	—	—	3	1	—	—
Follicular neoplasm cat. 5	—	—	2	—	—	1
Follicular neoplasm cat. 3	2 (d/l)	—	—	—	—	—
Follicular neoplasm cat. 2	6		—	—	—	—
Medullary ca. thyroid	—	—	—	2	—	—
Undifferentiated ca.	—	—	—	2	—	—
Hurthle cell neoplasm cat. 3	2	—	—	—	—	—
Granulomatous thyroiditis	1(d/l)	—	—	—	—	—

**Table 4:** Distribution of complications in both approaches.

Complications	Number of patients with medial approach	Number of patient with lateral approach	Improved after
Conversion to strap muscles cutting	3	0	—
External branch of SLN injury	5	0	Steroid administration
Recurrent laryngeal nerve injury (transient + permanent)	3	1	Steroid administration
Parathyroid injury	3	0	—
Seroma or hematoma formation	3	0	—
Swallowing difficulty	1	0	—
Postoperative stridor or respiratory distress	1	1	—
Tetany (clinical and biochemical)	3	0	Calcium supplements (Sr Ca monitoring)
Hypothyroidism (biochemical)	14	20	Thyroxine administration (TSH, T3, T4 monitoring)

## Data Availability

All data are mentioned in the tables. Any additional data can be made available upon request to the corresponding author.
